# Prescreening-Based Subset Selection for Improving Predictions of Earth System Models With Application to Regional Prediction of Red Tide

**DOI:** 10.3389/feart.2022.786223

**Published:** 2022-01-25

**Authors:** Ahmed S. Elshall, Ming Ye, Sven A. Kranz, Julie Harrington, Xiaojuan Yang, Yongshan Wan, Mathew Maltrud

**Affiliations:** 1 Department of Earth, Ocean, and Atmospheric Science, Florida State University, Tallahassee, FL, United States; 2 Center for Economic Forecasting and Analysis, Florida State University, Tallahassee, FL, United States; 3 Environmental Sciences Division and Climate Change Science Institute, Oak Ridge National Laboratory, Oak Ridge, TN, United States; 4 Center for Environmental Measurement and Modeling, United States Environmental Protection Agency, Gulf Breeze, FL, United States; 5 Fluid Dynamics and Solid Mechanics Group, Los Alamos National Laboratory, Los Alamos, NM, United States

**Keywords:** regional environmental management, harmful algae blooms of red tide, climate models and Earth system models, HighResMIP of CMIP6, multi-model ensemble methods, sub-ensemble selection and subset selection, decision-relevant metrics

## Abstract

We present the ensemble method of prescreening-based subset selection to improve ensemble predictions of Earth system models (ESMs). In the prescreening step, the independent ensemble members are categorized based on their ability to reproduce physically-interpretable features of interest that are regional and problem-specific. The ensemble size is then updated by selecting the subsets that improve the performance of the ensemble prediction using decision relevant metrics. We apply the method to improve the prediction of red tide along the West Florida Shelf in the Gulf of Mexico, which affects coastal water quality and has substantial environmental and socioeconomic impacts on the State of Florida. Red tide is a common name for harmful algal blooms that occur worldwide, which result from large concentrations of aquatic microorganisms, such as dinoflagellate *Karenia brevis*, a toxic single celled protist. We present ensemble method for improving red tide prediction using the high resolution ESMs of the Coupled Model Intercomparison Project Phase 6 (CMIP6) and reanalysis data. The study results highlight the importance of prescreening-based subset selection with decision relevant metrics in identifying non-representative models, understanding their impact on ensemble prediction, and improving the ensemble prediction. These findings are pertinent to other regional environmental management applications and climate services. Additionally, our analysis follows the FAIR Guiding Principles for scientific data management and stewardship such that data and analysis tools are findable, accessible, interoperable, and reusable. As such, the interactive Colab notebooks developed for data analysis are annotated in the paper. This allows for efficient and transparent testing of the results’ sensitivity to different modeling assumptions. Moreover, this research serves as a starting point to build upon for red tide management, using the publicly available CMIP, Coordinated Regional Downscaling Experiment (CORDEX), and reanalysis data.

## INTRODUCTION

To improve raw outputs directly given by Earth system models (ESMs) for providing useful services to societal decision making, a combination of multiple methods is often used such as bias-correction to account for systematic errors (Szabó-Takács et al., 2019; Wang et al., 2019), ensemble recalibration to improve ensemble characteristics (Manzanas et al., 2019), downscaling to improve the spatial and temporal resolution (Gutowski Jr. et al., 2016; Gutowski et al., 2020), and ensemble methods to select and combine different models. Ensemble methods are an active research area as multi-model ensemble can be more robust then a single-model ensemble (DelSole et al., 2014; Al Samouly et al., 2018; Wallach et al., 2018). Single model ensemble is a single Earth system model (ESM) with multiple realizations given perturbed parameters, initialization, physics, and forcings. Multi-model ensemble refers to an ensemble of multiple ESMs with single or multiple realizations of each ESM. Ensemble methods aim at selecting and combining multiple ESMs to form a robust and diverse ensemble of models. Ensemble methods include model weighting by assigning lower weights to less favorable models (Knutti, 2010; Weigel et al., 2010), bagging by using subsets of data or variables (Ahmed et al., 2019), subset-selection in which the best performing independent models are selected (Chandler, 2013; Herger et al., 2018; Ahmed et al., 2019; Hemri et al., 2020), and the combination of these methods (e.g., using subset selection prior to model weighting).

This study focuses on subset selection, which has not received adequate attention in climate and Earth system research (DelSole et al., 2013; Herger et al., 2018). In subset selection, a subset of models, which have better performance in a set of models, are selected as ensemble members. One model could perform better than other models due to more accurate parameterizations, higher spatial resolution, more tight calibration to relevant data sets, inclusion of more physical components, more accurate initialization, and imposition of more complete or more accurate external forcings (Haughton et al., 2015). In addition, one model could perform better than another model for a specific application as we show in this study. Accordingly, a question that often arises in multi-model combination is whether the original set of models should be screened such that “poor” models are excluded before model combination (DelSole et al., 2013). One argument is that combining all “robust” and “poor” models to form an ensemble (e.g., by assigning lower weights for poorly performing models than others) is an intuitive solution that has advantage over subset selection that uses the best performing model (Haughton et al., 2015). One justification is that, while the “poor” model can be useless by itself, it is useful when combined with other models due to error cancellation (Knutti et al., 2010; DelSole et al., 2013; Herger et al., 2018). Another justification is that no small set of models can represent the full range of possibilities for all variables, regions and seasons (Parding et al., 2020). On the other hand, it has been argued that the objective of subset selection is to create an ensemble of well-chosen, robust and diverse models, and thus if the subset contains a large enough number of the highest ranked and independent models, then it will have the characteristics that reflect the full ensemble (Evans et al., 2013).

Subset selection has several advantages and practical needs. First, a thorough evaluation is generally required to remove doubtful and potentially erroneous simulations (Sorland et al., 2020), and to avoid the least realistic models for a given region (McSweeney et al., 2015). Second, predictive performance can generally improve from model diversity rather than from larger ensemble (DelSole et al., 2014). A reason for this is that as more models are included in an ensemble, the amount of new information diminishes in proportion, which may lead to overly confident climate predictions (Pennell and Reichler, 2011). Accordingly, several studies (Herger et al., 2018; Ahmed et al., 2019; Hemri et al., 2020) developed evaluation frameworks in which subset selection is performed prior to model weighting. A third advantage of subset selection is to identify models based on physical relationships highlighting the importance of process-based model evaluation. For example, Knutti et al. (2017) defined the metric of September Arctic sea ice extent, showing that models that have more sea ice in 2100 than observed today and models that have almost no sea ice today are not suitable for the projection of future sea ice. There is no obvious reason to include these “poor model” that cannot simulate the main process of interest. Likewise, for our case study, we show that models that are unable to simulate the looping ofa regional warm ocean current in the Gulf of Mexico (i.e., Loop Current) are unsuitable for our environmental management objective (i.e., prediction of the harmful algal blooms of red tide) as described later. Yun et al. (2017) indicate that incorporating such process-based information is important for highlighting key underlying mechanistic processes of the individual models of the ensemble. Fourth, subset selection allows for flexibility in terms of metrics and thresholds to tailor the multi-model ensemble for the needs of specific applications (Bartók et al., 2019). As noted by Jagannathan et al. (2020), model selection studies are often based on evaluations of broad physical climate metrics (e.g., temperature averages or extremes) at regional scales, without additional examination of local-scale decision-relevant climatic metrics, which can provide better insights on model credibility and choice. For example, Bartók et al. (2019) and Bartók et al. (2019) employ subset selection to tailor the ensemble for energy sector needs, and local agricultural need in California, respectively. Finally, another practical need for subset selection is that, due to high computational cost, it is common that only a small subset of models can be considered for downscaling (Ahmed et al., 2019; Parding et al., 2020; Sorland et al., 2020).

Although there is a need for an efficient and versatile method that finds a subset which maintains certain key properties of the ensemble, few work has been done in climate and Earth system research (Herger et al., 2018). Without a well-defined guideline on optimum subset selection (Herger et al., 2018; Ahmed et al., 2019; Bartók et al., 2019; Parding et al., 2020), it is unclear how to best utilize the information of multiple imperfect models with the aim of optimizing the ensemble performance and reducing the presence of duplicated information (Herger et al., 2018). It may be difficult to predict exactly how many models are necessary to meet certain criteria, and subsets with good properties in one region are not guaranteed to maintain the same properties in other regions (Ross and Najjar, 2019). Typically, modelers make their own somewhat subjective subset choices, and use equal weighting for the models in the subset (Herger et al., 2018). A commonly used approach is model ranking, typically based on model performance to select the top models, which is generally the top three to five models (Jiang et al., 2015; Xuan et al., 2017; Hussain et al., 2018; Ahmed et al., 2019). For example, to derive an overall rank for each model, Ahmed et al. (2019) use comprehensive rating metric to combine information from multiple goodness-of-fit measures for multiple climate variables based on the ability to mimic the spatial or temporal characteristics of observations. Then to form the multi-model ensemble, Ahmed et al. (2019) select the four top-ranked models to evaluate the two cases of equal weighting and a bagging technique of random forest regression. A limitation of this approach is the arbitrary choice of the number of the top ranked model to include. For example, Ross and Najjar (2019) evaluate six subset-selection methods with respect to performance, and investigate the sensitivity of the results to the number of model chosen. They show that selection methods and models used should be carefully chosen. To aid this common approach of subset selection, Parding et al. (2020) present an interactive tool to compare subsets of CMIP5 and CMIP6 models based on their representation of the present climate, with user-determined weights indicating the importance of different regions, seasons, climate variables, and skill scores. This allows the users to understand the implications of their different subjective weights and ensemble member choices.

A less subjective approach for subset selection is to use a method that is designed to address specific key properties of the ensemble. In other words, a subset-selection method finds a subset which maintains certain key properties of the ensemble. Key properties include any combination of several criteria that are performance, ensemble range, ensemble spread, capture of extreme events, model independence, and decision relevant metrics. First, the performance criterion reflects the model’s skills in representing past and present climate and Earth system states. Examples include subset-selection methods to favor skilled models (Bartók et al., 2019), and to eliminate models with poorest representation of the present system states (Parding et al., 2020). A second criterion is the range of projected climate and Earth system changes. For example, McSweeney et al. (2015) developed a subset-selection method that captures the maximum possible range of changes in surface temperature and precipitation for three continental-scale regions. Third, the model spread criterion ensures that the ensemble contains representative models that conserve as much as possible the original spread in climate sensitivity and climate future scenarios with respect to variables of interest (Mendlik and Gobiet, 2016; Bartók et al., 2019). Fourth, another subset selection criterion, which is related to model spread, is the captures extreme events (Cannon, 2015; Mendlik and Gobiet, 2016; Farjad et al., 2019). Although some sectors are affected by mean climate changes, the most acute impacts are related to extreme events (Eyring et al., 2019). Fifth, model independence is another important criterion, which can be accounted for using diverse approaches. Sanderson et al. (2015) propose a stepwise model elimination procedure that maximizes intermodel distances to find a diverse and robust subset of models. Similarly, Evans et al. (2013) and Herger et al. (2018) use an indicator method with binary weights to find a small subset of models that reproduces certain performance and independence characteristics of the full ensemble. Binary weights are either zero or one for models to be either discarded or retained, respectively. Sixth, an additional criterion that is particularly important from many climate services is to consider regional application and decision-relevant metrics (Bartók et al., 2019; Jagannathan et al., 2020). Since a primary goal of climate research is to identify how climate affects society and to inform decision making, a community generally needs rigorous regional-scale evaluation for different impacted sectors that include agriculture, forestry, water resources, infrastructure, energy production, land and marine ecosystems, and human health (Eyring et al., 2019). By considering this criterion, subset-selection is not based on general model evaluation irrespective of the application (e.g., Sanderson et al., 2017), but is rather based on regional model evaluation with sector-specific information (Elliott et al., 2015). This includes, for example, considering a combination of climate hazards at a specific region (Zscheischler et al., 2018), and the use of application-specific metrics as in this study.

This study complements an important aspect of subset selection by explicitly considering application specific metrics for subset selection based on a prescreening step. To find more skillful and realistic models for a specific process or application, we develop an indicator-based subset-selection method with a prescreening step. In a prescreening step, models are scored based on physical relationships and their ability to reproduce key features of interest, highlighting the importance of process-based and application specific evaluation of climate models. Our method extends the indicator method based on binary weights of Herger et al. (2018), by scoring each model based on evolving binary weights, which are either zero or one for models to be either discarded or selected, respectively, as explained in the method section. Thus, irrespective of the general predictive performance of the model for the variables of interest (e.g., temperature, sea surface height, wind speed, and precipitation), the model performance is evaluated based on suitability to specific applications for a given problem definition with key features of interest.

In this case study of red tide, models that cannot reproduce key features of interest are the models that cannot simulate the process of Loop Current penetration into the Gulf of Mexico, for example, along with other key features as explained in the method section. Red tide is a common name of harmful algae blooms that occur in coastal regions worldwide due to high concentrations of marine microorganisms such as dinoflagellates, diatoms, and protozoans. Along the West Florida Shelf in the Gulf of Mexico, red tide occurs by the increase of the concentration of *Karenia brevis*, a toxic mixotrophic dinoflagellate. This study focuses on Loop Current (LC), which is one of the main drivers of red tide in the West Florida Shelf (Weisberg et al., 2014; Maze et al., 2015; Perkins, 2019). LC is a warm ocean current that penetrates and loops through the Gulf of Mexico until exiting the gulf to join the Gulf Stream. Several relations have been established between red tide and LC (Weisberg et al., 2014; Maze et al., 2015; Liu et al., 2016; Weisberg et al., 2019). The relation discussed in Maze et al. (2015) shows that the LC position, which can be inferred from sea surface heigh, can be a definitive predictor of a large red tide bloom possibility. Using CMIP6 and reanalysis data of sea surface heigh as described in the method section, we show that this prescreening-based subset-selection step can help reduce the ensemble size without degrading the predictive performance. We additionally illustrate the caveats of using non-representative models given the notation of error cancellation, showing that that a parsimonious ensemble can be more robust.

In the remainder of the manuscript, we present in *Methods* the red tide case study including the CMIP6 data, reanalysis data, and *Karenia brevis* data. *Methods* also presents the prescreening-based subset selection method. *Results* presents the results, which is following in *Discussion* by providing a discussion on subset selection, challenges of seasonal prediction, and the study limitations and outlook. Finally, we summarize our main findings, and draw conclusions in *Conclusion*.

## METHODS

### FAIR Guiding Principles

To better support transparency and reproducibility of scientific research, data and codes of scientific research should be part of the scholarly work, and must be considered and treated as a first-class research product (Horsburgh et al., 2020). We follow the FAIR Guiding Principles for scientific data management and stewardship (Wilkinson et al., 2016). Accordingly, the data and codes that are used and developed for this study are Findable, Accessible, Interoperable, and Reusable (FAIR). With respect to the “findable” criterion, our data and codes for data analysis are presented in Jupyter notebooks (Elshall, 2021) to provide rich metadata about the used CMIP data, reanalysis data and *Karenia brevis* data (*Data*). With respect to the “Accessible” criterion, the notebooks are opensource and are available on GitHub (Elshall, 2021). Additionally, the notebooks are supported by Colab cloud computing to make the codes immediately accessible and reproducible by anyone with no software installation and download to the local machine. With respect to the “interoperable” criterion, which refers to the exchange and use of information, the notebooks provide rich metadata with additional analysis details not found in the manuscript. This allows users to make use of the presented information by rerunning the codes to reproduce the results, and to understand the sensitivity of the results to different assumptions and configurations as described in the manuscript. Also, the codes can be used to visualize additional data and results that are not shown in the manuscript as described below. With respect to the “reusable” criterion, all the used data are publicly available, and the codes have publicly data usage license. This allows the users to build additional components to the codes as discussed in the manuscript.

### Data

**FIGURE 1 |:**
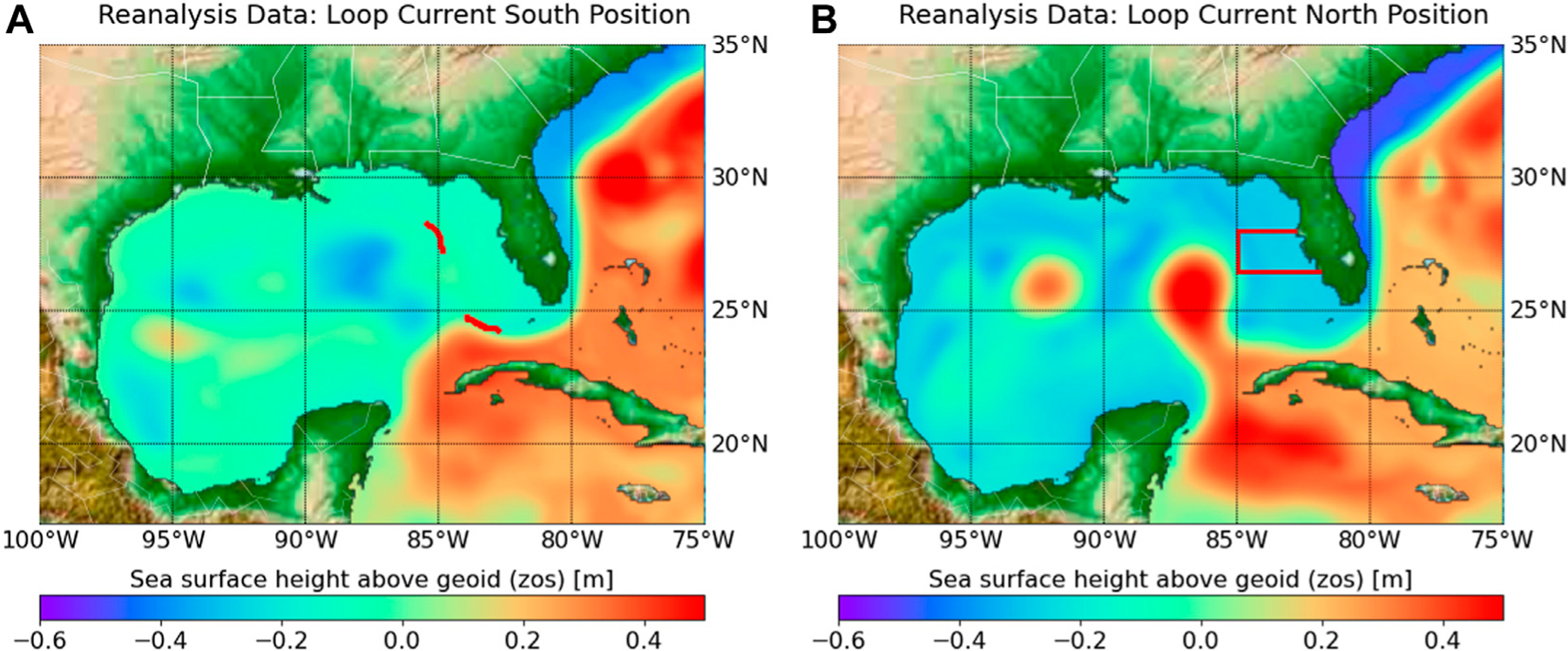
Observation reanalysis data of sea surface height above geoid (zos) [m] showing **(A)** LC-S and **(B)** LC-N. Two red segments along the 300 m isobath in **(A)** are used to determine Loop Current position. The area where red tide blooms are considered by Maze et al. (2015) and this study is shown in the red box of **(B)**.

The *Karenia brevis* cell count used in this study are from the harmful algal bloom database of the Fish and Wildlife Research Institute at the Florida Fish and the Wildlife Conservation Commission (FWRI, 2020). In the study area (Figure 1) and given the study period from 1993–01 to 2014–12, we identify 15 time intervals of large blooms, and 29 time intervals with no bloom; each time interval is six-month long. Following Maze et al. (2015), to identify a bloom/no-bloom event (*z*_*t*_), a large bloom is defined as an event with the cell count exceeding 1×10^5^ cells/L for ten or more successive days without a gap of more than five consecutive days, or 20% of the bloom length. Similar to Maze et al. (2015) we define no bloom as the absence of large bloom. The notebook “*Karenia*_*brevis_*data_processing” (Elshall, 2021) provides the data processing details.

We use global reanalysis data, which combine observations with shortrange weather forecast using weather forecasting models to fill the gaps in the observational records. We use the Copernicus Marine Environment Monitoring Service (CMEMS) monthly gridded observation reanalysis product. Th product identifier is Global_Reanalysis_PHY_001_030 (Drévillon et al., 2018; Fernandez and Lellouche, 2018), and can be download from Mercator Ocean International as part of the Copernicus Programme (https://resources.marine.copernicus.eu/products). The used CMEMS reanalysis product is a global ocean eddy-resolving reanalysis with approximatively 8 km horizontal resolution covering the altimetry from 1993 onward. Similar to CMIP6 data, we only focus on sea surface height above geoid, which is the variable name zos according to the Climate and Forecast Metadata Conventions (CF Conventions).

We use 41 CMIP6 model runs from 14 different models developed by eight institutes (Roberts et al., 2018, Roberts et al., 2019; Cherchi et al., 2019; Golaz et al., 2019; Held et al., 2019; Voldoire et al., 2019; Chang et al., 2020; Haarsma et al., 2020). CMIP6 data can be download from any node (e.g., https://esgf-data.dkrz.de/search/cmip6-dkrz) of the Earth System Grid Federation (ESGF) of World Climate Research Programme (WCRP). The study period is from 1993–01 to 2014–12. We select CMIP6 model runs from the historical experiment (Eyring et al., 2016) and the hist-1950 experiment (Haarsma et al., 2016), which are sibling experiments that use historical forcing of recent past until 2015. The historical simulation that starts from 1850 uses all-forcing simulation of the recent past (Eyring et al., 2016). The hist-1950 experiment that starts from 1950 uses forced global atmosphere-land simulations with daily 0.25° sea surface temperature and sea-ice forcings, and aerosol optical properties (Haarsma et al., 2016). For high-resolution models, our selection criteria are to select all model runs with gridded monthly “sea surface height above geoid,” which is the variable name zos according to the Climate and Forecast Metadata Conventions (CF Conventions), with nominal resolution less than or equal to 25 km. For each model we only consider variable zos. Given the available CMIP6 data until September 2020 when this study started, this resulted in 33 model runs. We mainly focus on high-resolution models with eddy-rich ocean resolution, which is important for simulating Loop Current. For our analysis purpose, we include two models with standard resolution. One is EC-Earth3P with nominal ocean resolution of about 100 km given in the hist-1950 experiment with three model runs, and E3SM-1–0 with variable ocean resolution of 30–60 km given in the historical experiment with five model runs.

### Model Independence

To account for model independence, we use institutional democracy (Leduc et al., 2016), which can be regarded as a first proxy to obtain an independent subset (Herger et al., 2018), reflecting a priori definition of dependence. For the same institution we created further subsets for different grids. This is the case for the standard- and medium-resolution models of EC-Earth-Consortium that use ORCA1 and ORCA025 grids, respectively. It is also the case for the high-resolution and medium-resolution model of MOHC-NERC that uses ORCA12 and ORC025 grids, respectively. The ORCA family is a series of global ocean configurations with tripolar grid of various resolutions. Thus, the considered 14 models that are listed aphetically by model name in Table 1, results in 11 independent model subsets.

For each independent model subset (IMS), multiple perturbed runs of (parameter) realizations (r), initializations (i), physics (p), and forcings (f) are considered. For example, IMS01 has only one model run r1i1p1f1, and IMS11 has seven model runs, three with perturbed initialization r1i (1–3)p1f1, and four with perturbed parameter realizations r (1–4)i1p1f3 as shown in Table 1. Note that this naming convention are relative given different modeling groups. For example, the coupled E3SM-1–0 simulations (Golaz et al., 2019) use five ensemble members that are r (1–5)i1p1f1 representing five model runs with different initialization. Each ensemble member (i.e., independent model subset, IMS) in Table 1 contains one or more models, and each model has one or more model runs. These model runs of each ensemble member should not simply be included in a multi-model ensemble as they represent the same model, hence artificially increasing the weight of models with more model runs. On the other hand, using only one model run per ensemble member discards the additional information provided by these different runs (Brunner et al., 2019). Accordingly, the zos data of each ensemble member is averaged in the way described in *Loop Current Position and Karenia brevis Blooms*.

With the default model independence criteria of institutional democracy and ocean grid we identify 11 ensemble members listed in Table 1. The notebook “SubsetSelection” (Elshall, 2021) and its interactive Colab version (https://colab.research.google.com/github/aselshall/feart/blob/main/i/c2.ipynb) provide other model independence criteria that can be investigated by the users. For example, a second case is to use institutional democracy criterion as the first criterion, ocean grid as a second criterion and experiment as a third criterion, which results in 13 ensemble members. In this case historical experiment and hist-1950 experiment are assumed to be independent. A third case is to assume all models are independent, which results in 14 ensemble members. A fourth case is to assume all models are independent, and use experiment as a second criterion, which results in 16 ensemble members. A fifth case is to assume that all members are independent, which results is 41 ensemble members. The code additionally allows for any user defined criteria. While the presented results in this paper are all based on the default model independence criteria, the user can instantly use the above link to investigate the sensitivity of the prescreening and subset selection results and reproduce all figures and under different model independence criteria.

### Loop Current Position and *Karenia brevis* Blooms

The mechanisms of initiation, growth, maintenance, and termination of red tides have not been fully understood. Yet Loop Current, which is a warm ocean current that moves into the Gulf of Mexico, is an important factor that controls the occurrence of red tide (Weisberg et al., 2014; Maze et al., 2015; Perkins, 2019). Maze et al. (2015) shows that the difference between time intervals of large blooms and no blooms is statistically significant for the Loop Current’s position. Maze et al. (2015) also show that the Loop current in a north position penetrating through the Gulf of Mexico is a necessarily condition for a large Karenia brevis bloom to occur. As such, when the Loop Current is in the south position shown in Figure 1A, which is hereinafter denoted as Loop Current-South (LC-S), then there is no large bloom (Maze et al., 2015). When the Loop Current is in the north position shown in Figure 1B, which hereinafter is denoted as Loop Current-North (LC-N), then there could be either large blooms or no blooms. This relationship between the loop current positions and Karenia brevis is based on retention time. With approximately 0.3 divisions per day, Karenia brevis is a slow growing dinoflagellate that requires an area with mixing slower than the growth rate to form a bloom (Magaña and Villareal, 2006). As such, LC-N increases the retention rate allowing bloom formation, if other conditions are ideal (Maze et al., 2015). While there are several studies that establish different relationships between Loop Current and Karenia brevis (Weisberg et al., 2014; Maze et al., 2015; Liu et al., 2016; Weisberg et al., 2019), the aim of this study is not to support or refute any of these relationships, but to use the study of Maze et al. (2015) for the purpose of our subset selection analysis.

The LC and its eddies can be detected from sea surface height variability. When the difference between the average sea surface height of the north and south segments along the 300 m isobath (Figure 1A) is positive and negative, this is a good proxy for identify LC-N and LC-S, respectively (Maze et al., 2015). The zos data processing steps to determine the Loop Current positions (i.e., LC-N and LC-S) are as follows:

1. The zos data is preprocessed for the north and south segments (Figure 1A) for all model runs and observation analysis data. Model runs and observation reanalysis data are sampled using nearest neighborhood method along the line points (approximately spaced at 1 km interval between two neighboring points) of the north and south segments (Figure 1A). The nearest neighborhood sampling is performed using the python package of xarray project (http://xarray.pydata.org) that handles NetCDF (Network Common Data Form) data formats with file extension NC that is used typically for climate data (e.g., CMIP and reanalysis data). This has an additional practice advantage of reducing the size of the ESMs and reanalysis data. For example, in this case preprocessing CMIP6 and CMEMS data reduced that data size from more than 80 GB to about 11 MB interactive cloud computing feasible. Given data preprocessing, we have a zos datum *h*_(*j,k,l,m,n,t*)_ for a model run with index *j*, an ensemble member with index *k*, a spatial point along the segment with index *l*, a segment (i.e., the north or south segment in Figure 1A) with index *m*, a model and reanalysis datasets temporal interval (i.e., 1 month) with index *n*, and a prediction interval with index *t*.
2. The expectation of zos data is taken for all model runs *j* ∈ [1*, J*] of each ensemble member *M*_*k*_
(1) $$h_{k,l,m,n,t}=E_{j}\left(h_{j,k,l,m,n,t}|M_{k}\right)$$
  The size *J* of each ensemble member varies depending on the number of model runs in the ensemble member, with the minimum *J* = 1 for ensemble member IMS01 and the maximum *J* = 7 for ensemble member IMS11 (Table 1).
3. The zos data is averaged for all ensemble members *k* ∈ [1*,K*]
(2) $$h_{l,m,n,t}=E_{k}\left(E_{j}\left(h_{j,k,l,m,n,t}|M_{k}\right)\right)$$
  where *k* is the index of each ensemble member *M*_*k*_. The size *K* of the multi-model ensemble varies based on subset selection (*Prescreening*), which determines the inclusion and exclusion of ensemble members. For example, using all available ensemble members without any subset selection results in *K* = 11 that is all the independent model subsets in Table 1. If we evaluate *k* for only one ensemble member for prescreening purpose (*Prescreening*), then *K* = 1.
4. For each of the north and south segments the expected zos is calculated for each segment
(3) $$h_{m,n,t}=E_{l}\left[E_{k}\left(E_{j}\left(h_{j,k,l,m,n,t}|M_{k}\right)\right)\right]$$
5. The zos data of the north segment is subtracted from the south segment
(4) $$h_{n,t}=\Delta_{m}\left[E_{l}\left[E_{k}\left(E_{j}\left(h_{j,k,l,m,n,t}|M_{k}\right)\right)\right]\right]$$
  resulting in zos difference data *h*_*n,t*_ with *n* ∈ [1*, N*] and *t* ∈ [1*, T*]. As such, *N* represents the interval length such that *N* = 3 for a season interval, and *N* = 6 for a semiannual interval, and *T* represents the number of intervals. For example, given *N* = 6 as considered in this study and the 22-year study period, then *T* = 44.
6. The maximum *h*_*n,t*_ in the 6-month interval is selected to obtain the zos anomaly per time interval
(5) $$h_{t}=\underset{h_{n}}{\max}\left(\Delta_{m}\left[E_{l}\left[E_{k}\left(E_{j}\left(h_{j,k,l,m,n,t}|M_{k}\right)\right)\right]\right]\right)$$

For each zos anomaly datum *h*_*t*_, positive and negative values are used as an indicator of LC-N dominated interval and LC-S dominated interval, respectively. Selecting the maximum value $\underset{h_{n}}{\max}\left(.\right)$ is more robust than using the average value, which may dilute the signals since the Loop Current position is a cycling event, recalling that loop current has a random and chaotic cycle with the average period of 8–18 months per cycle (Sturges and Evans, 1983; Maze et al., 2015).

The objective of this analysis is not to model the LC cycle, but rather to use the relationship between Loop Current position and *Karenia brevis* bloom of Maze et al. (2015) to obtain a heuristic coarse-temporal-resolution relation between Loop Current position and Karenia brevis. Thus, the *h*_*t*_ values given by Eq. 5 can be expressed as an indicator function for LC-N:

(6) $$H_{LC-N}\left(h_{t}\right)=\{\begin{array}{c} 1,\;h_{t}\geq 0\\ 0,\;h_{t}<0 \end{array}$$

and LC-S:

(7) $$H_{LC-S}\left(h_{t}\right)=\{\begin{array}{c} 1,\;h_{t}<0\\ 0,\;h_{t}\geq 0 \end{array}$$

such that $H_{LC-N}(h_{t})=1$ and $H_{LC-S}(h_{t})=1$ indicate a LC-N interval and LC-S interval, respectively. Eqs 6 and 7 are convenient to use since we are not interested in the value of zos anomaly between the north and south segments per se, but rather in sign difference. Finally, Eqs 5–7 are valid for both model simulation and observation reanalysis data, which hereinafter are donated as *h*_*t*_ and *h*_*t,obs*_, respectively.

### Model Performance Metrics

**FIGURE 2 |:**
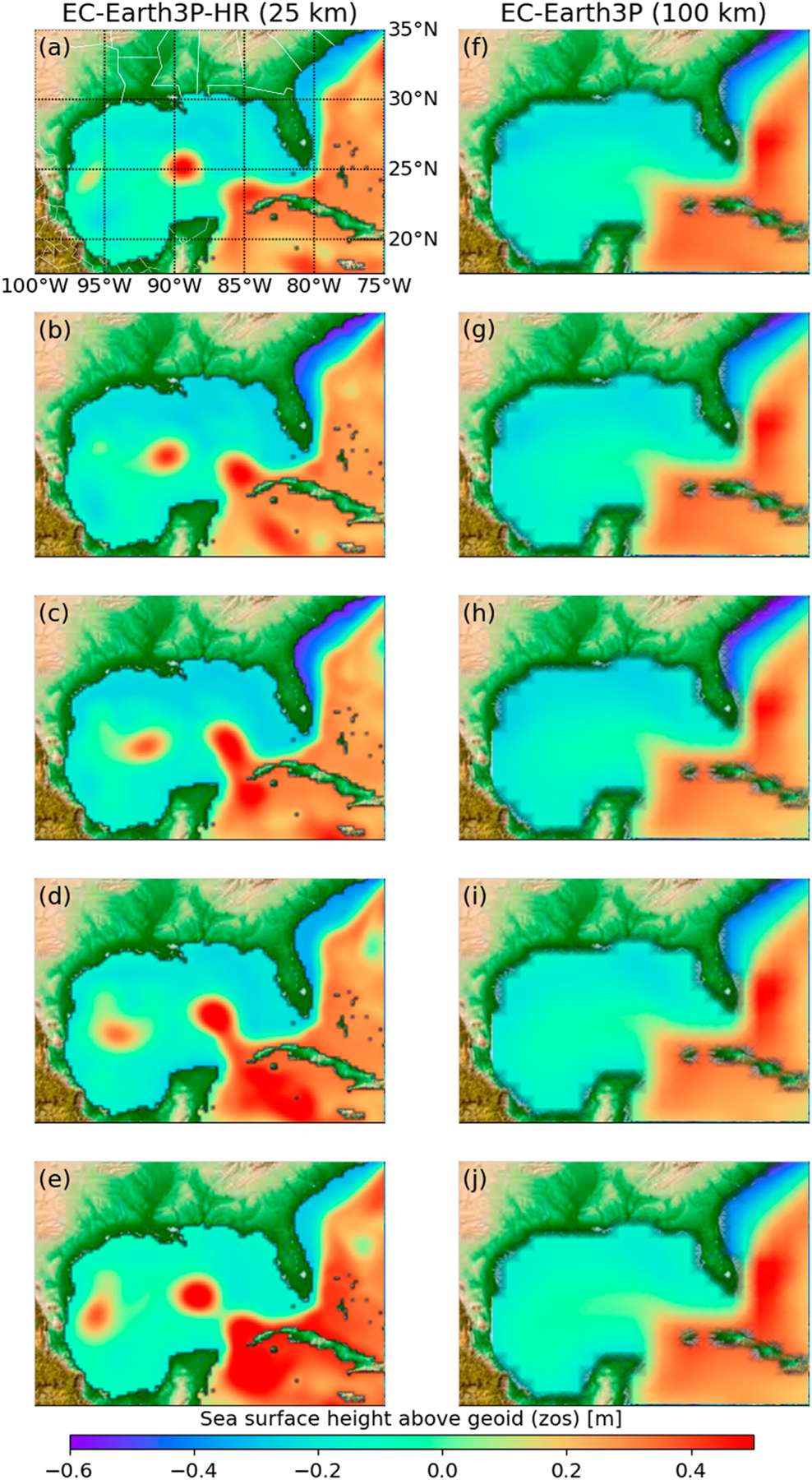
Snapshots of sea surface height above geoid (zos) [m] from 1993–02 to 1993–06 simulated using **(A–E)** a high-resolution ESM, and **(F–J)** standard-resolution ESM with nominal resolution of 10 and 100 km, respectively.

A model performance is based on its ability to reproduce the observed phenomena. We define three qualitative metrics to prescreen for physical relationships, and four quantitative metrics of the model performance. Based on this prescreening we can do subset selection. For prescreening, a process-based metric is needed, for example, to understand if the model can simulate certain mechanistic aspects of the problem of interest. For example, Christensen et al. (2010) use metrics that capture aspects of model performance in reproducing large-scale circulation patterns and meso-scale signals. A qualitative metric reflects if the model is suitable or unsuitable for reproducing key features of the problem. In our case study, models that cannot reproduce key features of interest would be the models that cannot 1) simulate the penetration of LC into the Gulf of Mexico, 2) represent the alternation of LC in the North and South positions given the empirical method (Eqs 5–7), 3) reproduce the higher frequency of Loop Current in the northern and southern positions as described below. For example, with respect to (1), the Loop Current penetrates the Gulf of Mexico extending its northward reach with eddy shedding as shown by the high-resolution model EC-Earth3P-HR (Figures 2A–C). As such, intrusion of cooler water increases the stratification of the core of the Loop Current, and the Loop Current becomes unstable forming anticyclonic eddy that breaks from the parent Loop Current westward without reconnecting (Caldwell et al., 2019), as shown by the high-resolution model EC-Earth3P-HR (Figures 2D,E). On the other hand, the standard-resolution model EC-Earth3P (Figures 2F–J) cannot reproduce the observed physical phenomena, and thus unsuitable for this application. Models that are unable to simulate LC-N are unsuitable for this environmental management purpose. Justifications about selecting these three qualitative metrics and details about them are given below. Finally, for a further illustration of the models that are capable and incapable of reproducing the Loop Current, Elshall (2020) shows an animation of a Loop Current cycle of year 2010 given monthly zos data for all the 41 model runs in Table 1 shown side-by-side with the reanalysis data. In addition, the reader can visualize the reanalysis data in Figure 1 and the CMIP6 data in Figure 2 for any month in the study period 1993–2015 using the Jupyter notebook “DataVisualization_zos” (Elshall, 2021), and its interactive Colab version (https://colab.research.google.com/github/aselshall/feart/blob/main/i/c1.ipynb).

The binary qualitative metrics (*y*_1_-*y*_3_) used for prescreening are as follows:

#### Physical phenomena simulation (y_1_):

Accurate simulation of Loop Current positions is generally a challenging task, yet the objective of this first metric is to determine if the model can simulate LC-N irrespective of the accuracy. Thus, the model receives a score one *y*_1_ = 1 if it can simulate LC-N (e.g., Figures 2A–E), and zero *y*_1_ = 0 otherwise (e.g., Figures 2F–J), i.e.,

(8) $$y_{1}=\{\begin{array}{l} 1,\;\;\sum_{t=1}^{T}H_{LC-N}\left(h_{t}\right)>0\\ 0,\;\sum_{t=1}^{T}H_{LC-N}\left(h_{t}\right)=0 \end{array}$$

such that $\sum_{t=1}^{T}H_{LC-N}(h_{t})$ is the count on LC-N intervals given the total number of intervals *T* = 44 as explained before.

#### Oscillating event representation (y_2_):

This metric is specific to the method of Maze et al. (2015) for determining LC-N and LC-S. If the sea surface height is consistently higher at the north segment than at the south segment, then the model is unable to represent alternation of LC-N and LC-S according to the proxy method of Maze et al. (2015). In this case, the model receives a score zero *y*_2_ = 0, and one *y*_2_ = 1 otherwise, i.e.,

(9) $$y_{2}=\{\begin{array}{l} 1,\;\;0<\sum_{t=1}^{T}H_{LC-N}\left(h_{t}\right)<T\\ 0,\;\;\;\;\sum_{t=1}^{T}H_{LC-N}\left(h_{t}\right)=T \end{array}$$

#### Oscillating event realism (y_3_):

If the frequency of LC-N is greater than that of LC-S for a model, the model receives the score of one *y*_3_ = 1 and zero *y*_3_ = 0 otherwise, i.e.,

(10) $$y_{3}=\{\begin{array}{l} 1,\;\;\sum_{t=1}^{T}H_{LC-N}\left(h_{t}\right)\geq \sum_{t=1}^{T}H_{LC-S}\left(h_{t}\right)\\ 0,\;\sum_{t=1}^{T}H_{LC-N}\left(h_{t}\right)<\sum_{t=1}^{T}H_{LC-S}\left(h_{t}\right) \end{array}$$

It is more realistic that the frequency of LC-N is greater than that of LC-S. In the study of Maze et al. (2015), the ratio of the LC-S intervals $\sum_{t=1}^{T}H_{LC-N}(h_{t})$ to the total number of intervals *T* = 60 is 0.267, given their altimetry data product with study period of 15 years and 3-month interval (i.e., *N* = 3). In this study the ratio of LC-S to total number of intervals is 0.273, given our reanalysis product with *T* = 44 and *N* = 6 as previously explained.

We define four quantitative metrics (*y*_4_-*y*_7_) to evaluate the predictive performance, and the scoring rules (*y*_8_) to evaluate complexity. These performance criteria are as follows.

#### Oscillating event frequency (y_4_):

This is the ratio of the number of a LC position (LC-S or LC-N) to the total number of intervals. Hereinafter, we refer to the oscillating event frequency as the number of LC-S to the total number of intervals *T*,

(11) $$y_{4}=\frac{\sum_{t=1}^{T}H_{LC-S}\left(h_{t}\right)}{T}$$

which can be compared to reanalysis data that is 0.273 as presented in the results section. Additionally, we define the oscillating event frequency error as

(12) $$y_{4,err}=\frac{\left|\sum_{t=1}^{T}H_{LC-S}\left(h_{t}\right)-\sum_{t=1}^{T}H_{LC-S}\left(h_{t,obs}\right)\right|}{T}$$

which is the absolute difference of LC-S counts of ensemble prediction *h*_*t*_ and reanalysis data *h*_*t,obs*_.

#### Temporal match error (y_5_):

This is a temporal match of model predictions and reanalysis data with respect to LC position for LC-N

(13) y5,LC−N=∑t=1THLC−N(ht,obs)−∑t=1T(ht,obs≥0^ht≥0)∑t=1THLC−N(ht,obs)

for LC-S

(14) y5,LC−S=∑t=1THLC−S(ht,obs)−∑t=1T(ht,obs<0^ht<0)∑t=1THLC−S(ht,obs)

and both positions

(15) y5=T−∑t=1T(ht,obs≥0^ht≥0)−∑t=1T(ht,obs<0^ht<0)T

such that $\sum_{t=1}^{T}H_{LC-N}\left(h_{t,obs}\right)$ and $\sum_{t=1}^{T}H_{LC-S}\left(h_{t,obs}\right)$ are the counts of the LC-N and LC-S intervals, respectively, given the observation reanalysis data *h*_*t,obs*_; the terms ∑t=1T(ht,obs≥0^ht≥0) and ∑t=1T(ht,obs<0^ht<0) are the temporal match counts of model simulation and reanalysis data for LC-N and LC-S, respectively. The logical conjunction ∧ gives a value of one when the statement (ht,obs≥0^ht≥0) is true if *h*_*t,obs*_ ≥ 0 and *h*_*t*_ ≥ 0 are both true, otherwise gives a value of zero if false. Temporal match is the most challenging task. While ESMs are well established on climate timescale, the temporal match at seasonal timescale can be challenging (Hewitt et al., 2017). Generally speaking, the hist-1950 and historical experiments are free-running, and accordingly are neither designed nor expected to have temporal coincide with real-world conditions, which is especially true for the historical experiment. However, one aim of this study is to investigate if any temporal match is possible given the used heuristic relation for determining Loop Current position with a coarse temporal resolution of 6-month interval.

#### Karenia brevis error (y_6_):

A false negative prediction of *Karenia brevis* bloom occurs when large bloom coincides with LC-S. For the study period, we define the *Karenia brevis* error as the ratio of the number of LC-S with large bloom to the number of large-bloom *N*_*bloom*_

(16) y6=∑t=1T(ht<0^H(zt)=1)Nbloom

where *H*(*z*_*t*_) is an indicator function with one and zero for large bloom and no bloom, respectively.

#### Root-mean-square error (y_7_):

It is the root-mean-square error (RMSE) between model simulation and reanalysis data

(17) $$y_{7}=\sqrt{\frac{\sum_{t=1}^{T}{\left(h_{t}-h_{t,obs}\right)}^{2}}{T}}$$

The defined metrics (*y*_1_- *y*_7_) are specifically designed to judge the predictive performance of these ESMs with respect to the targets of a specific application, and are not meant to judge the predictive skill of these ESMs globally or regionally for general purposes. Judging the predictive skills of these models with respect to global or regional simulations of sea surface height above geoid (variable: zos) or any other variable, is beyond the scope of this work.

### Prescreening

**FIGURE 3 |:**
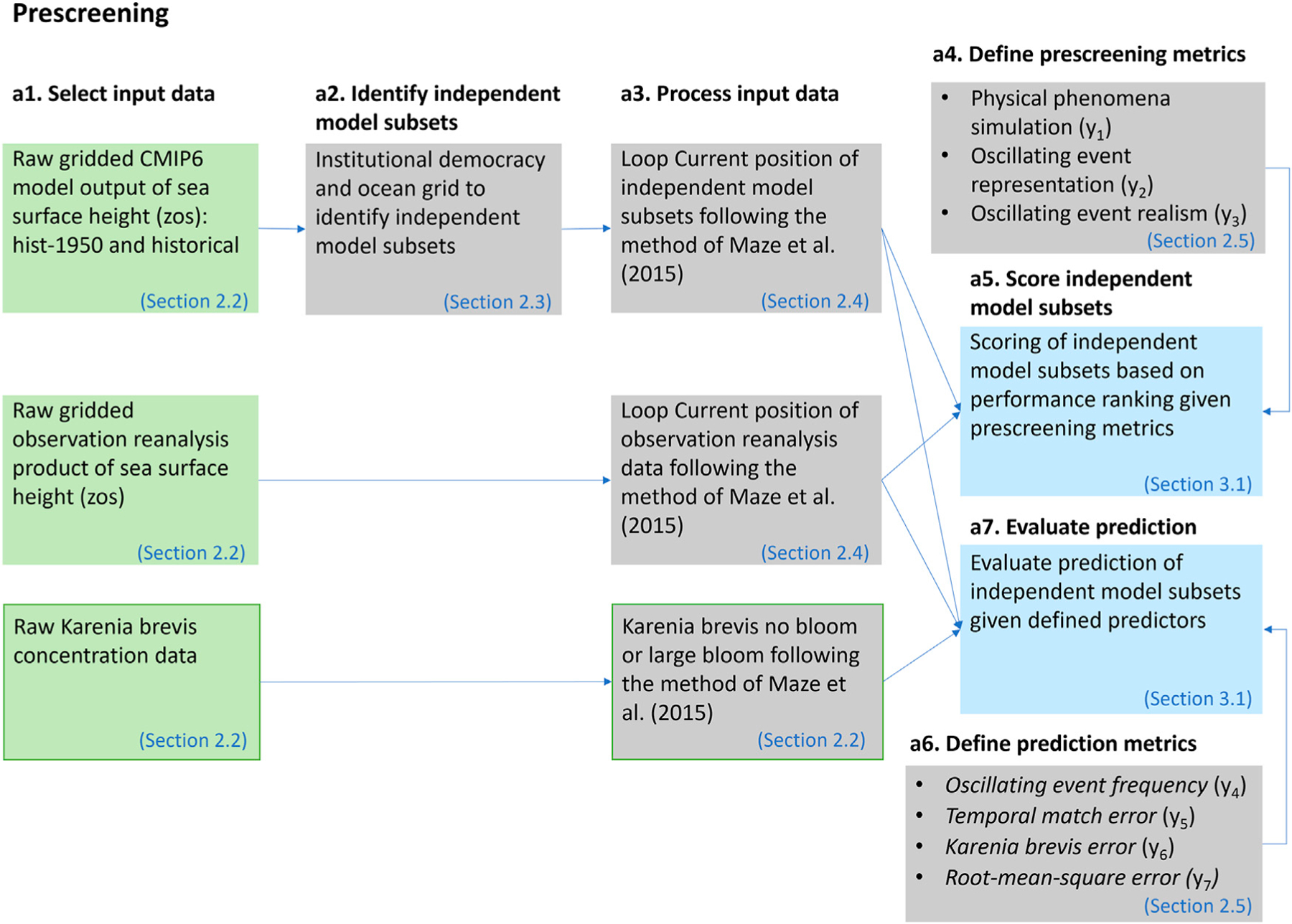
The prescreening method.

Evaluation of specific regional applications is another important criterion, which is the focus of this manuscript. We develop a subset-selection method that extends the binary method of Herger et al. (2018) based on a prescreening step as shown in Figure 3. Model independence is accounted for as described in *FAIR Guiding Principles*, and a score is obtained for each ensemble member using three binary qualitative metrics *y*_1_-*y*_3_ (*Model Independence*). Binary refers to a score of either zero or one if the ensemble member is unable or able to produce the metric target. The three binary metrics (Eqs 8–10) are evolving such that if the ensemble member fails the first metric, then it will consequently fail in the other two, and will accordingly receive a score of zero. For example, given score (*y*_1_, *y*_2_, *y*_3_), the model receives a score from zero to three for score (0,0,0), (1,0,0), (1,1,0), and (1,1,1), respectively. In other words, if a model score is one for *y*_3_ (Eq. 10) it will by default score ones for *y*_1_ (Eq. 11) and *y*_2_ (Eq. 9).

### Subset Selection

**FIGURE 4 |:**
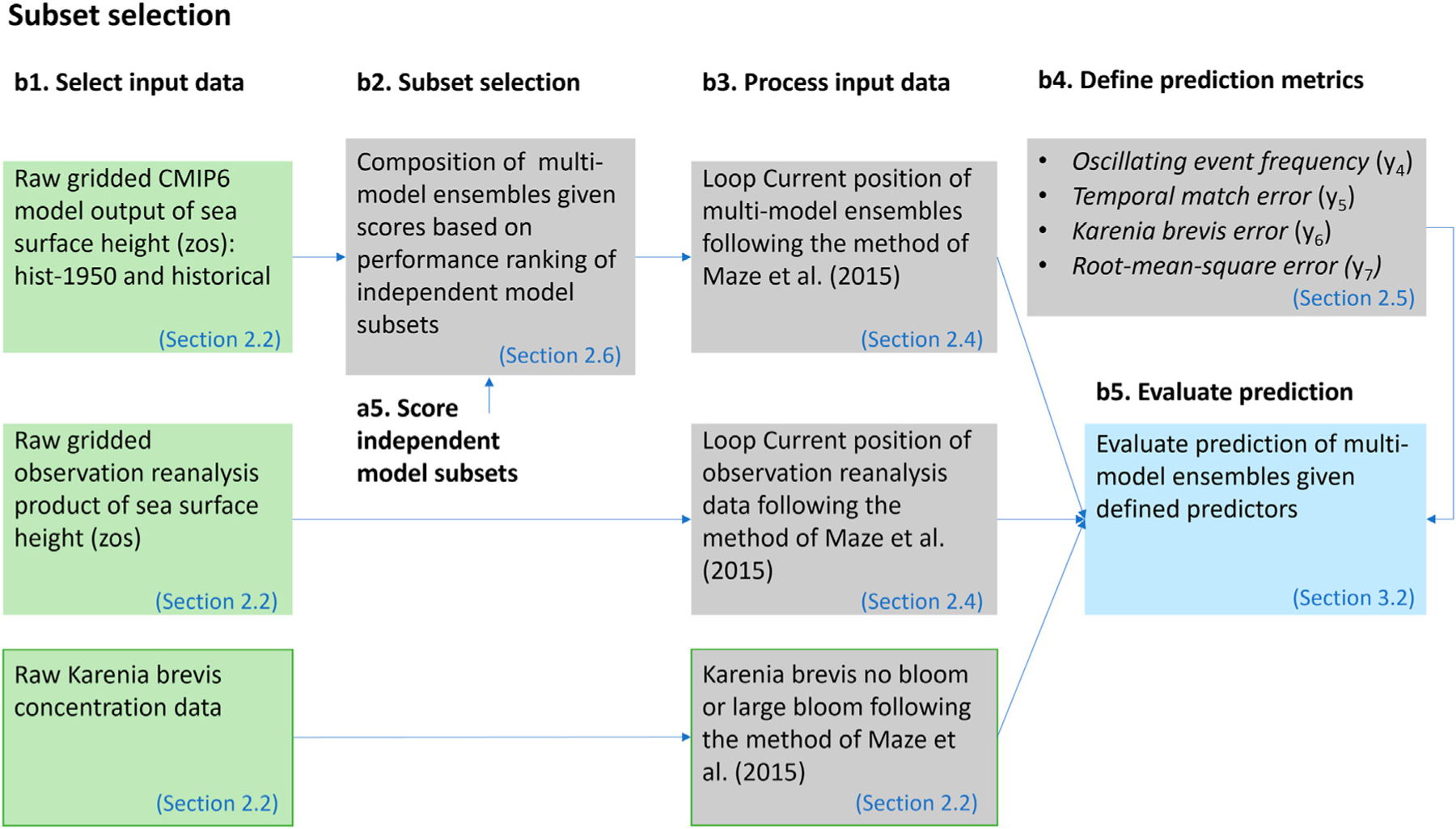
The subset-selection method.

The subset selection step is shown in Figure 4. In this step we compose five multi-model ensembles using simple-average multi-model ensemble (SME). Each SME is composed of ensemble members based on prescreening score. The notation SME3210 means that members with prescreening score from zero to three are included in the ensemble. The notation SM321X means that members with prescreening score from one to three are included in the ensemble and members with prescreening score of zero are excluded, and so on. Ensemble SME321X, SME32XX, and SME3XXX exclude ensemble members based on the three binary qualitative metrics (*y*_1_- *y*_3_), respectively. These are evolving metrics such that if an ensemble member scores zero in *y*_1_, it will score zero in *y*_2_ and *y*_3_, and have an overall score of zero. If a model has a score *y*_3_ = 1, it will by default score one in *y*_1_ and *y*_2_, and have an overall score of three. As such, SME3210 contains all ensemble members with scores from zero to three, which is all the 11 ensemble members listed in Table 1. On the other hand, SME3XXX contains the best ensemble members, which are the ones with a score of three. Ensemble SME32XX contains ensemble members with scores of three and two, and so on. On the other hand, ensemble SMEXXX0 contains only the least performing ensemble members with a score of zero. More discussion on the model scores is given in the next section. We evaluate the predictive performance of these five multi-model ensembles using the quantitative metrics (*y*_4_-*y*_7_). The evaluation of these five multi-model ensembles serves multiple purposes as described in the results section.

## RESULTS

### Prescreening

**FIGURE 5 |:**
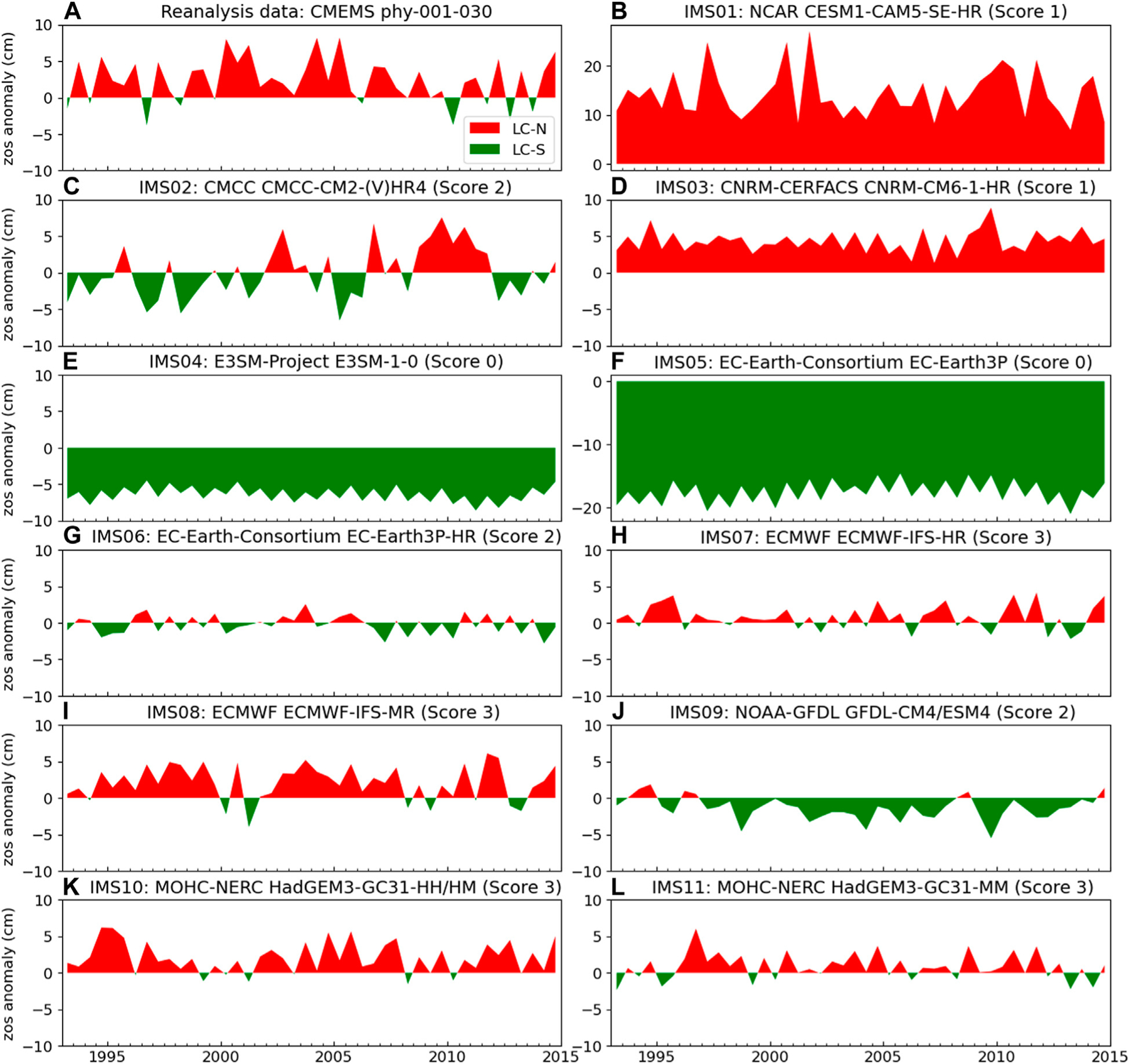
The surface height above geoid (zos) anomaly (Eq. 5) of **(A)** reanalysis data, and **(B–L)** enesmble members (i.e, independent model subsets). The title of the reanalysis data shows the data provider name, and product ID. The title of ensemble member shows ensemble member number, modeling group name, model name(s), and ensmble member score.

We plot the oscillation of the Loop Current position for each ensemble member (Figure 5), following the zos data processing steps described in *Loop Current Position and Karenia brevis Blooms*. This is to conduct qualitative comparison between the reanalysis data (Figure 5A) and the prediction of each ensemble member (Figures 5B–L). Accordingly, we score the ensemble member given its performance with respect to three binary evolving metrics (*y*_1_-*y*_3_). The score is zero if the ensemble member fails to pass all the three metrics. This is the case for E3SM-1–0 of DOE-E3SM-Project (Figure 5E) and the EC-Earth3P of EC-Earth-Consortium (Figure 5F). As these two ensemble members do not pass the first metric of physical phenomena simulation (*y*_1_) that is the simulation of the LC-N, then accordingly they score zero in the next two metrics of oscillating event representation (*y*_2_) and oscillating event realism (*y*_3_). This is not unexpected as these two ensemble members are standard-resolution ESMs, which do not have improved process description as the high-resolution ESMs do. The standard-resolution grids EC60to30 of E3SM-1–0 and ORCA1 of EC-Earth3P do not explicitly resolve the mesoscale eddies and boundary currents, but rather require global parametrization of mesoscale eddies. For example, EC60to30 is an eddy closure (EC) grid with global parameterization that is not designed to resolve regional spatial phenomena. On the other hand, with a high horizontal resolution, the eddy-permitting grids such as eORCA12, ORCA12, eORCA025, and ORCA025 (Table 1) can resolve mesoscale eddies, and do not require ocean eddy flux parameterization. For comparison of high- and standard-resolution grid see also Figure 2. On the other hand, the model runs of CESM1-CAM5-SE-HR of NCAR (Figure 5B) and CNRM-CM6–1-HR of CNRM-CERFACS (Figure 5D) can simulate LC-N, but without a sign difference of zos at the two segments (Figure 1A), and accordingly fail in the second metric of oscillating event representation (*y*_2_). These two ensemble members receive a score of one. This score does not indicate that the sea surface height simulation of these models is poor in general, but rather that these models are unsuitable for this target given the problem definition. The ensemble members of CMCC-CM2-(V)HR4 of CMCC (Figure 5C), EC-Earth3P-HR of EC-Earth-Consortium (Figure 5G), and GFDL-CM4/ESM4 of NOAA-GFDL (Figure 5J), pass the second metric, but fail on the oscillating event realism (*y*_3_). These ensemble members show a higher LC-S frequency than LC-N, which is not consistent with the reanalysis data (Figure 5A). Accordingly, these three ensemble members receive a score of two. Finally, the ensemble members that pass the three evolving binary metrics and receive a score of three are ECMWF-IFS-HR of ECMWF (Figure 5H), ECMWF-IFS-MR of ECMWF (Figure 5I), HadGEM3-GC31-HH/HM of MOHC-NERC (Figure 5K), and HadGEM3-GC31-MM of MOHC-NERC (Figure 5L). Visual inspection shows that these four ensemble members are qualitatively similar to the reanalysis data (Figure 5A) with respect to Loop Current position oscillation.

Using metrics *y*_4_–*y*_7_, we evaluate the predictive performance of these 11 ensemble members with respect to reanalysis data as shown in Table 2. According to Maze et al. (2015) there are no red tide blooms for LC-S, and there are either large blooms or no blooms for LC-N. The results of our reanalysis data shown in Table 2 are consistent with Maze et al. (2015) such that none of the 12 intervals of LC-S has large blooms for the study period. Out of the 32 intervals of LC-N, 15 intervals have large blooms. This indicates that LC-N is a necessarily condition for the large bloom to occur and be sustained. Given the reanalysis data, the LC-S frequency is 0.273 for our 22-year study period, which is comparable to Maze et al. (2015), which is 0.267 for their 15-year study period. The ensemble members IMS07, IMS10, IMS11, and IMS08 have the best agreement with the reanalysis data showing LC-S frequencies (*y*_4_) of 0.295, 0.318, 0.205, and 0.182, respectively. These correspond to the oscillating event frequency errors (*y*_4*,err*_) of 0.022, 0.045, −0.068, and −0.091, respectively. Ensemble members that can simulate the oscillation of LC-N and LC-S and have the best temporal match are IMS08, IMS07, IMS10, and IMS11 with temporal match error (*y*_5_) of 27, 34, 34, and 41%, respectively. Given the high-resolution model runs, IMS08, IMS07, IMS10, and IMS11 have the lowest *Karenia brevis* error (*y*_6_) of 0.1, 0.3, 0.3, and 0.3, respectively. IMS09, IMS08, IMS10, IMS03 have the lowest RMSE (*y*_7_) of 3.77, 3.87, 3.88, and 4.02, respectively. While no ensemble member is consistently ranked as the top ensemble member given the four metrics, IMS08 is ranked twice as the top ensemble member given the two metrics *y*_5_ and *y*_6_. Thus, this analysis shows that there is no single ensemble member that consistently perform better with respect to all metrics, and that different ensemble members show both over and underestimation of zos anomaly. These two remarks indicate the importance of using a multi-model ensemble.

### Subset Selection

**FIGURE 6 |:**
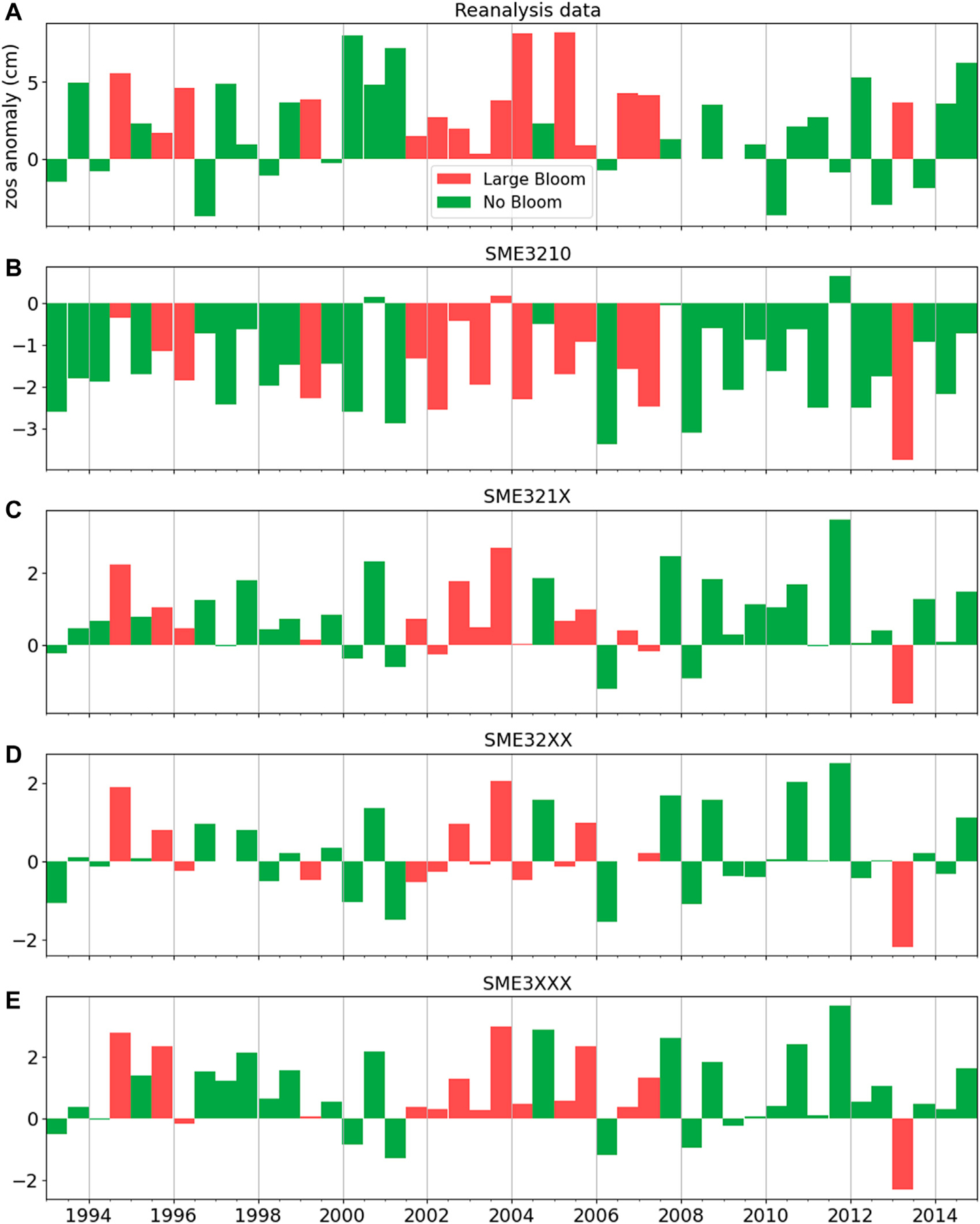
Temporal match of large bloom/no bloom with Loop Current positions given the surface height above geoid (zos) anomaly (Eq. 5) of **(A)** reanalysis data, and **(B–E)** simulations of four multi-model ensembles. Positive and negative bars indicate Loop Current North **(LC-N)** and Loop Current South (LC-S), respectively.

There is generally no specific guideline on the composition of multi-model ensemble of ESMs. While composing information from multiple imperfect ensemble members can be an arbitrarily task, the prescreening step can help find subsets that maintain key features of the problem of interest. We first discuss the two ensembles of SME3210 and SME321X. The ensemble SME3210, which includes both high- and standard-resolution model runs, is generally a flawed ensemble composition, since we know from prior existing knowledge of other studies (Caldwell et al., 2019; Hoch et al., 2020) that standard-resolution ESMs are generally incapable of simulating Loop Current. On the other hand, SME321X is the most straightforward ensemble composition that acknowledges prior information, and includes all high-resolution runs that are capable of simulating Loop Current. We consider SME321X as our reference ensemble. Figure 6 shows the predictive performance of the four multi-model ensembles. Large red tide blooms do not occur for LC-S given reanalysis data (Figure 6A). Comparing reanalysis data (Figure 6A) and the multi-model ensembles (Figures 6B–E) shows that ensembles based on prior information (i.e., SME321X, SME32XX, and SME3XXX) correspond better to reanalysis data than without accounting for prior information (i.e., SME3210).

Visual examination in Figure 6 is insufficient to understand the impact of prescreening information (i.e., SME32XX and SME3XXX) in comparison to the reference ensemble SME321X without prescreening information, and qualitative metrics are needed. Table 3 quantitatively shows that including standard-resolution model runs (i.e., SME3210) results in prediction degradation with respect to the four qualitative metrics (*y*_4_-*y*_7_). As can be calculated from raw data in Table 3, SME321X shows relatively good agreement with the reanalysis data with a LC-S frequency (*y*_4_) of 0.227, temporal match error (*y*_5_) of 36%, *Karenia brevis* bloom error (*y*_6_) of 20%, and RMSE (*y*_7_) of 3.71.

Another approach for ensemble composition is to use information from the prescreening step. These are ensembles SME32XX and SME3XXX that exclude the models that cannot represent the oscillation of LC-N and LC-S (*y*_2_). Ensemble SME3XXX only includes model runs with realistic presentation of LC-N and LC-S (*y*_3_). SME32XX shows degraded predictions with respect to the reference ensemble SME321X for all the four quantitative metrics (*y*_4_-*y*_7_). This is not unexpected since members of SME321X show both under and overestimation. For simple model average of model runs with over and underestimation the errors are expected to cancel out (Herger et al., 2018). However, this is not the case for SME3XXX that leverages on most information gained from the prescreening step (i.e., by only including the best members that meet the targets of interest). SME3XXX shows mixed predictive performance with respect to the reference ensemble showing better performance with respect to temporal match error (*y*_5_) of 25% (versus 36% for the reference ensemble), *Karenia brevis* error (*y*_6_) of 13% (versus 20% for the reference ensemble), and RMSE (*y*_7_) of 3.68 (versus 3.71 for the reference ensemble), but inferior performance with respect to LC-S frequency (*y*_4_) of 0.205 (versus 0.273 and 0.227 for the reanalysis data and reference ensemble, respectively). Yet temporal coverage error is not important for future predictions as discussed in *Discussion*. The relatively good performance of SME3XXX is expected, because this ensemble ensures that members with good performance are only included.

Table 3 additionally shows the case of SMEXXX0, which only considers standard-resolution runs. SMEXXX0 shows a poor predictive performance with respect to all metrics. We present the SMEXXX0 ensemble to illustrate the breakthrough of the HighResMIP of CMIP6. With respect to sea surface height simulation and regional phenomena, our results clearly show the significant improvement of the high-resolution runs of CMIP6 in comparison to the standard-resolution models that are typical to CMIP5.

### Ensemble Composition

**FIGURE 7 |:**
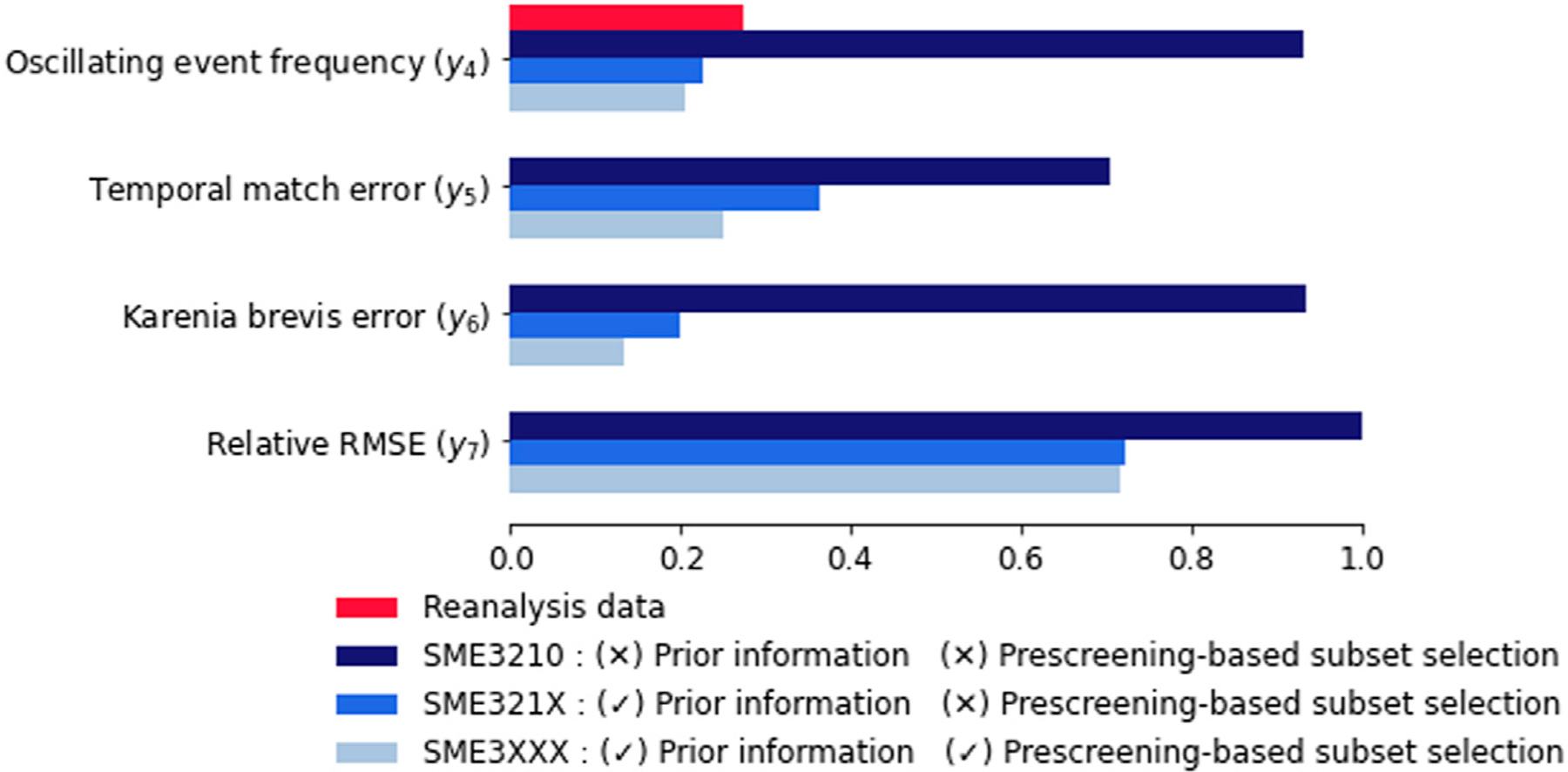
Predictive performance (*y*_4_–*y*_7_) given different ensemble composition criteria.

Our results show that using prior information is important for ensemble composition, and prescreening-based subset selection can be helpful. Figure 7 summarizes the effect of different ensemble composition criteria. Prior information appears as an important criterion that should be considered as SME3210 has the worst predictive performance with respect to the other ensembles given *y*_4_–*y*_7_. Prescreening-based subset selection seems to relatively improve the predictive performance given *y*_5_–*y*_7_, and slightly degraded performance with respect to *y*_4_. However, pre-screening-based subsect selection has a second conceptual advantage. Given prior information, the first approach of using all the available ensemble members (i.e., SME321X) is a straightforward choice that can result in error cancellation. The second approach of using information from prescreening results in a reduced size ensemble (i.e., SME3XXX), which maintains the most important ensemble characteristics with respect to the problem of interest. While in the first approach we attempt to maintain a more conservative ensemble, with the second approach we create an ensemble with robust ensemble members. Our results suggest that pre-screening based subset section used to substitute or prior to model weighting, which is a subject of a future research.

## DISCUSSION

### Subset Selection

To find a robust ensemble that improves the predictive performance of ESMs, this article shows the importance of subset selection based on prior information, prescreening, and process-based evaluation. By evaluating the prescreening-based subset-selection method we deduce two key points as follows. First, we present additional advantages to subset selection that are not well recognized in the literature, which is the importance of subset selection based on process-based evaluation similar to Yun et al. (2017). Eliminating models from an ensemble can be justified if they are known to lack key mechanisms that are indispensable for meaningful climate projections (Weigel et al., 2010). As shown in this study, models that cannot simulate the processes of interest based on a prescreening step can be excluded from the ensemble without degrading the ensemble prediction. Second, the selection of subset-selection method depends on the criteria that are relevant for the application in question (Herger et al., 2018). For example, the process-based evolving binary weights developed in this study is particularly important to eliminate non-representative models. Unlike other subset-selection methods in literature that can be technically challenging to implement, we present a subset-selection method that can be frequently used, as it is intuitive and straightforward to apply. This approach is an addition to subset-selection literature, and is not meant to supersede any of the existing approaches in the literature.

### Seasonal Prediction Limitations

Improving seasonal prediction of ESMs to provide useful services for societal decision making is an active research area. Techniques to improve temporal correspondence between predictions and observations at the regional scale is needed for climate services in many sectors such as energy, water resources, agriculture, and health (Manzanas et al., 2019). In this study we used raw outputs without using a postprocessing method to improve temporal correspondence of seasonal prediction. Our results show that the temporal correspondence is not poor, which could be just coincident. Alternatively, this could be attributed to the chosen Loop Current position heuristic with a coarse-temporal-resolution. Accordingly, given a long 6-month period, this is not a month-by-month or season-by-season temporal match, but rather a pseudo-temporal correspondence that captures the general pattern of a dynamic process. Accordingly, using this heuristic relationship, a form of temporal relationship might be possible as long as there is no large drift. If such a temporal correspondence cannot be established for ESMs for Loop Current or other factors that drives the red tide, this would limit the use of the ESMs in terms of providing an early warning system. However, this will not affect the main purpose of the intended model, which is to understand the frequency and trend of red tide under different climate scenarios and estimating the socioeconomic impacts accordingly. If temporal correspondence is required, seasonal prediction of ESMs has generally been possible through statistical and dynamical downscaling methods, and other similar techniques such as pattern scaling and use of analogue (van den Hurk et al., 2018). Alternatives to more complex statistical downscaling techniques to improve temporal correspondence include bias correction (Rozante et al., 2014; Oh and Suh, 2017; Wang et al., 2019), ensemble recalibration (Sansom et al., 2016; Manzanas et al., 2019), and postprocessing techniques such as copula-based postprocessing (Li et al., 2020). For example, to improve temporal correspondence of seasonal prediction, Manzanas (2020) use bias correction and recalibration methods to remove mean prediction bias, and intraseasonal biases from drift (i.e., lead-time dependent bias).

### Limitations and Outlook

In this study we present the advantages of subset selection using Loop Current prediction as an example. We show these advantages for the simplest case of using a deterministic analysis, and by considering only historical data. For red tide management purpose, which is to understand the frequency of red tide and the corresponding socioeconomic impacts under different climate scenarios, further steps are needed. First, using CMIP6 model projection data is important to understand the frequency and future trends of red tide under different Shared Socioeconomic Pathways (SSPs) of CMIP6 in which socio-economic scenarios are used to derive emission scenarios without mitigation (i.e., baseline scenario) and with mitigation (i.e., climate polices). Additionally, CMIP6 data can be readily replaced by high resolution data of Coordinated Regional Downscaling Experiment (CORDEX) as soon as they become available. CORDEX which is driven by the CMIP outputs, provides dynamically downscaled climate change experiments for selected regions (Gutowski Jr. et al., 2016; Gutowski et al., 2020). Second, we need to extend our method to a probabilistic framework that considers both historical and future simulations. As historical assessment criteria are not necessarily informative in terms of the quality of model projections of future climate change, identifying the performance metrics that are most relevant to climate projections is one of the biggest challenges in ESM evaluation (Eyring et al., 2019). As the choice of model is a tradeoff between good performance in the past and projected climate change, selecting only the best performing models may limit the spread of projected climate change (Parding et al., 2020). Exploring such trade-off is warranted in a future study in which a probabilistic framework (e.g., Brunner et al., 2019) is needed to account for model performance, model independence, and the representation of future climate projections. Third, it is imperative to consider not only Loop Current, but also other factors that control red tide such as alongshore and offshore wind speed, African Sahara dust, and atmospheric CO2 concentration need to be considered. To account for these different factors simultaneously to predict red tide, machine learning is needed similar to the study of Tonelli et al. (2021) that uses CMIP6 data and machine learning to study marine microbial communities under different climate scenarios. In summary, there are still many further steps needed to develop a probabilistic machine learning framework for regional environmental management of red tide using ESMs of CMIP6 and CORDEX when available. This study is merely a showcase for the potential of using ESMs for red tide management.

## CONCLUSION

To improve ensemble performance and to avoid prediction artifacts from including non-representative models, which are models that cannot simulate the process(es) of interest, we introduce a prescreening based subset-selection method. Including non-representative models with both over and underestimation can result in error cancellation. Whether to include or exclude these non-representative models from the ensemble is a point that requires further investigation through studying model projection. We present a generic subset-selection method to exclude non-representative models based on process-based evolving binary weights. This prescreening step screens each model with respect to its ability to reproduce certain key features. This research emphasizes the importance of ensemble prescreening, which is a topic that is rarely discussed. The presented subset-selection method is flexible as it scores each model given multiple binary criteria. This allows the user to systematically evaluate the sensitivity of the results to different choices of ensemble members. Such flexibility is generally needed to allow the user to understand the implication of ensemble subset selection under different cases (e.g., historic versus historic and future simulations, etc.). Our prescreening-based subset selection method is not meant to replace any of the existing approaches in the literature, but to provide a straightforward and easy-to-implement approach that can be used for many climate services in different sectors as needed.

## Figures and Tables

**TABLE 1 |:** Independent model subsets based on institutional democracy and using ocean grid as a secondary criterion when applicable.

Independent model subset (IMS)	Institution	Country	Model (reference)	Experiment ID	Members	Ocean model resolution	Ocean model	Ocean grid	ESM nominal resolution (km)
IMS01	NCAR	United States	CESM1-CAM5-SE-HR (Chang et al., 2020)	hist-1950	r1i1p1f1	0.1° (11 km) nominal resolution	POP2	POP2-HR	25
IMS02	CMCC	Italy	CMCC-CM2-HR4 (Cherchi et al., 2019)	hist-1950	r1i1p1f1	0.25° from the Equator degrading at the poles	NEMO v3.6	ORCA025	25
			CMCC-CM2-VHR4 (Cherchi et al., 2019)	hist-1950	r1i1p1f1	0.25° from the Equator degrading at the poles	NEMO v3.6	ORCA025	25
IMS03	CNRM-CERFACS	France	CNRM-CM6–1-HR (Voldoire et al. (2019))	hist-1950	r (1–3) i1p1f2	0.25° (27–28 km) nominal resolution	NEMO v3.6	eORCA025	25
			CNRM-CM6–1-HR (Voldoire et al., 2019)	Historical	r1i1p1f2	0.25° (27–28 km) nominal resolution	NEMO v3.6	eORCA025	25
IMS04	DOE-E3SM-Project	United States	E3SM-1–0 (Golaz et al., 2019)	Historical	r (1–5) i1p1f1	60 km in mid-latitudes and 30 km at the equator and poles	MPAS-O	EC60to30	100
IMS05	EC-Earth-Consortium	Europe	EC-Earth3P (Haarsma et al., 2020)	hist-1950	r (1–3) i1p2f1	about 1° (110 km)	NEMO v3.6	ORCA1	100
IMS06	EC-Earth-Consortium	Europe	EC-Earth3P-HR (Haarsma et al., 2020)	hist-1950	r (1–3) i1p2f1	about 0.25° (27–28 km)	NEMO v3.6	ORCA025	25
IMS07	ECMWF	Europe	ECMWF-IFS-HR (Roberts et al., 2018)	hist-1950	r (1–6) i1p1f1	25 km nominal resolution	NEMO v3.4	ORCA025	25
IMS08			ECMWF-IFS-MR (Roberts et al., 2018)	hist-1950	r (1–3) i1p1f1	25 km nominal resolution	NEMO v3.4	ORCA025	25
IMS09	NOAA-GFDL	United States	GFDL-CM4 (Held et al., 2019)	Historical	r1i1p1f1	0.25° (27–28 km) nominal resolution	MOM6	tri-polar grid	50
			GFDL-ESM4 (Held et al., 2019)	Historical	r (2–3) i1p1f1	0.25° (27–28 km) nominal resolution	MOM6	tri-polar grid	50
IMS10	NERC	United Kingdom	HadGEM3-GC31-HH (Roberts et al., 2019)	hist-1950	r1i1p1f1	8 km nominal resolution	NEMO v3.6	ORCA12	10
	MOHC-NERC	United Kingdom	HadGEM3-GC31-HM (Roberts et al., 2019)	hist-1950	r1i (1–3) p1f1	25 km nominal resolution	NEMO v3.6	ORCA12	50
IMS11	MOHC	United Kingdom	HadGEM3-GC31-MM (Roberts et al., 2019)	hist-1950	r1i (1–3) p1f1	25 km nominal resolution	NEMO v3.6	ORCA025	100
			HadGEM3-GC31-MM (Roberts et al., 2019)	Historical	r (1–4) i1p1f3	25 km nominal resolution	NEMO v3.6	ORCA025	25

**TABLE 2 |:** Raw data of Loop Current at North (LC-N) and South (LC-S) positions, and their relation to the occurrence of large blooms for reanalysis data, and each ensemble member (i.e., independent model subset, IMS). The ensemble size is the number of model runs per ensemble member, and the reanalysis data has only one realization. Note given Score (*y*_1_, *y*_2_, *y*_3_) the model receives a score from 0 to 3 for Score (0, 0, 0), Score (1,0,0), Score (1, 1, 0), and Score (1, 1, 1), respectively.

IMS	Ensemble	Count		Count LC-N		Count LC-S		Temporal match			RMSE	Score
	Size	LC-N	LC-S	No-Bloom	Large-Bloom	No-Bloom	Large-Bloom	LC-N	LC-S	Total		
Reanalysis data	1	32	12	17	15	12	0	32	12	44	0	3
IMS01	1	44	0	29	15	0	0	32	0	32	13.16	1
IMS02	2	20	24	14	6	15	9	15	7	22	5.48	2
IMS03	4	44	0	29	15	0	0	32	0	32	4.02	1
IMS04	5	0	44	0	0	29	15	0	12	12	9.27	0
IMS05	3	0	44	0	0	29	15	0	12	12	20.16	0
IMS06	3	20	24	13	7	16	8	13	5	18	4.34	2
IMS07	6	31	13	21	10	8	5	24	5	29	3.77	3
IMS08	3	36	8	22	14	7	1	28	4	32	3.87	3
IMS09	3	8	36	6	2	23	13	5	9	14	5.06	2
IMS10	4	35	9	24	11	5	4	26	3	29	3.88	3
IMS11	7	30	14	20	10	9	5	22	4	26	4.08	3

**TABLE 3 |:** Raw data of Loop Current at North (LC-N) and South (LC-S) positions, and their relation to the occurrence of large blooms simple-average multi-model ensemble (SME). The ensemble size refers to the number of model runs per multi-model ensemble.

SME	Ensemble	Count		Count LC-N		Count LC-S		Temporal match			RMSE
	size	LC-N	LC-S	No-Bloom	Large-Bloom	No-Bloom	Large-Bloom	LC-N	LC-S	Total	
Reanalysis data	1	32	12	17	15	12	0	32	12	44	0
SME3210	41	3	41	2	1	27	14	2	11	13	5.13
SME321X	33	34	10	22	12	7	3	25	3	28	3.71
SME32XX	28	23	21	17	6	12	9	17	6	23	3.92
SME3XXX	20	35	9	22	13	7	2	28	5	33	3.68
SMEXXX0	8	0	44	0	0	29	15	0	12	12	13.52

## Data Availability

The datasets presented in this study can be found in online repositories. The names of the repository/repositories and accession number(s) can be found below: Elshall, A.S. (2021). Codes for the article of prescreening-based subset selection for improving predictions of Earth system models for regional environmental management of red tide (v1.0). Zenodo. https://doi.org/10.5281/zenodo.5534931.
